# Prediction of Chloride Penetration Depth in Concrete Using a Combined Ensemble–Neural Network Architecture: Facing Data Saturation

**DOI:** 10.3390/ma19102118

**Published:** 2026-05-18

**Authors:** Changhwan Jang, So-Hee Kim, Yeong-Wi Jo, Hong-Gi Kim

**Affiliations:** 1 Graduate School of DNA Plus Convergence Technology, Daejin University, 1007 Hoguk-ro, Pocheon-si 11159, Republic of Korea; cjang@daejin.ac.kr (C.J.); ksh09@korea.kr (S.-H.K.); jonion@korea.kr (Y.-W.J.); 2 Construction Technology Examination Division, Ministry of Intellectual Property, 189 Cheongsa-ro, Daejeon 35208, Republic of Korea; 3 Port Development Division, Ministry of Oceans and Fisheries, 14 Jungang-Daero, Busan 48798, Republic of Korea

**Keywords:** machine learning, chloride penetration depth, hybrid architecture, neural networks, ensemble models

## Abstract

**Highlights:**

- The combination of architectures can lead to rapid data saturation.
- Simple architectures can actually be more effective with limited data.
- The hybrid model established a high precision, but not meaningfully in this study.
- The simple architecture provided efficient and compact results rather than the hybrid model.
- The method for improving accuracy, even with limited datasets, should be studied.

**Abstract:**

Chloride penetration depth (CPD) is a critical durability indicator for concrete structures, yet experimental data are often limited. This study evaluates whether increasing model complexity is beneficial under such constraints by comparing six machine learning and deep learning models—extreme gradient boosting, categorical boosting (CATB), random forest, multilayer perceptron (MLP), deep neural network (DNN), and a hybrid model combined with CATB and DNN (CatDNN)—using a dataset of 1078 cases. During training, CatDNN exhibited the earliest stabilization, reaching the best epoch at 40, while MLP and DNN stabilized after approximately 30 epochs. However, overfitting tracking revealed a flat tendency near 40 epochs for CatDNN, indicating potential data saturation. The test results showed small performance differences among all the models. CatDNN achieved the lowest max error (1.21), demonstrating effective residual correction, but its R^2^ (0.9123) was slightly lower than that of DNN (0.9129), suggesting that increased complexity did not yield meaningful improvement. The validation results confirmed high reliability across all the models (R^2^ ≥ 0.88). Overall, the findings indicate that, under limited data conditions, simple and well-fitted models can outperform or match complex hybrid architectures, emphasizing the importance of model efficiency over structural complexity.

## 1. Introduction

Chloride penetration is a critical deterioration factor in concrete structures. Inside the concrete, elements such as capillary cracks and interfacial transition zones act as pathways for chloride penetration. Due to the aging of coastal concrete structures around the world, rebars are corroded, and maintenance costs are increasing enormously [1]. In addition, in countries that use de-icing agents for snow removal, serious chloride damage can be observed in urban areas as well [2]. It is very fatal not only for the rebar, but also for the concrete itself. The main de-icing agent is calcium chloride. When calcium chloride meets free water inside the concrete, it releases heat and forms calcium oxychloride at the same time as the chemical reaction [3]. Calcium oxychloride is an expansive product that causes internal expansion and damage to concrete [4]. It is the same for rebar. The volume of rebar expands inside the concrete due to corrosion, causing internal expansion tensile force. As a result, the concrete is destroyed and the rebar is exposed, which causes an acceleration of damage [5]. Due to this significance, many studies have been conducted to preliminarily detect chloride penetration or predict depth [6]. In recent days, the most studied field is the prediction of chloride penetration depth (CPD) using artificial intelligence (AI) technology.

Before the AI era, prediction of CPD mainly relied on theoretical modeling [7], empirical trends [8], and statistical methods [9]. Each conventional method has shown high accuracy, but they were customized models for a specific condition. However, with the release of AI technology, if there is enough data, it is possible to classify the environment and conditions into classes to make more accurate predictions. In other words, it is now possible to classify any condition to reflect a variety of environments and conditions. The most representative technologies are machine learning (ML) and deep learning (DL). DL is a method of learning data using neural networks (NNs), which are mainly the backbone of AI technology. Because ML includes statistical modeling such as regression analysis, it is understood in a broader scope than DL. The most representative ML methods include random forest (RF), extreme gradient boost (XGB), categorical boost (CATB), support vector machine (SVM), and decision tree (DT). There are various types of neural networks used in DL; therefore, it is best to use NNs according to the purpose. Taffese et al. [10] performed a prediction of chloride durability grade with 843 observations collected. The study includes DT, SVM, and RF. As a result, SVM, RF, and another model, not DT, showed high accuracy [10]. In a similar case, Taffese et al. [11] predicted the chloride penetration coefficient of concretes using ML including XGB, RF, and DT. The results of Taffese et al. [11] indicated the lowest root-mean-square error (RMSE) and the best performance for predicting the chloride penetration coefficient. XGB is an ensemble model with improved performance over gradient boosting models, and it can be expected to have improved performance over other tree-based models. In addition to these representative examples, there are many similar studies [12,13,14].

There are DL studies in a more specific way. It predicts the CPD utilizing NNs. Wu et al. [15] predicted chloride penetration (CP) with time-dependent data. The utilized NNs in the study of Wu et al. [15] were bidirectional long short-term memory (Bi-LSTM), convolutional neural network (CNN), gate recurrent unit (GRU), and LSTM. Several NNs were used, but the most compact and typical NN, LSTM, showed the best results. Li et al. [16] predicted the CP coefficients of concrete using artificial neural networks (ANNs), also known as multi-layer perceptron (MLP), RF, and SVM. The results of Li et al. [16] indicated that the RF-SVM-ANN stacked state showed superior performance compared to using ANN solely. Liu et al. [17] used an ANN to deeply estimate the CP coefficient. Liu et al. [17] obtained 949 data cases and used 12 parameters such as water–cement ratio (W/C), exposure dates, curing time, and so on. By assigning the ANN into three roles, a strategy was established so that each purpose-specific ANN could complement the other ANNs. As a result, the prediction achieved a high accuracy of 0.95 determination coefficient (R^2^) value [17]. The application methods of NNs are limitless. Beyond the introduced studies of NN application cases, numerous other studies have employed various ways to estimate CPD using NNs [18,19,20,21].

Despite the wide range of NN applications and ML methods, the majority of studies still focus on comparing prediction performance to a single NN and other ML/DL models. Some studies aim to improve accuracy by increasing methodological complexity through combinations of different approaches. The idea that combining architectures can enhance prediction accuracy is reasonable. However, when only a limited dataset is available, it is unclear whether increasing model complexity is truly efficient. Many studies combine NNs or increase complexity to estimate parameters or specific target values. Yet, in cases like this study, where obtainable experimental data is limited, it is difficult to secure a sufficiently large dataset, and the analysis often relies on experimental results or information collected from the literature. In general, probability-based approaches are a powerful way to fill such gaps [22,23]. Nevertheless, it is still necessary to investigate whether combining limited data with higher NN complexity is an efficient strategy. This study aims to explore this research gap by using the CPD dataset, which is one of the challenging datasets to obtain experimentally in concrete research.

This study collected a total of 1078 CDP data cases, which are summarized in Table A1 of Appendix A. Eight input parameters were used: W/C, cement ratio, coarse aggregate ratio (Cagg), fine aggregate ratio (Fagg), admixture ratio (Adm), SiO_2_ ratio (SR), CaO ratio (CR), and immersion days (IMDY). The output parameter was the CPD. This study performed CPD estimation using three NN models and three ML models. The NN models included a hybrid architecture combining CATB and a deep neural network (DNN), referred to as CatDNN, DNN and MLP. For ML models, the widely used models XGB, CATB, and RF were employed.

## 2. Methodology

### 2.1. Research Process

**Figure 1:**
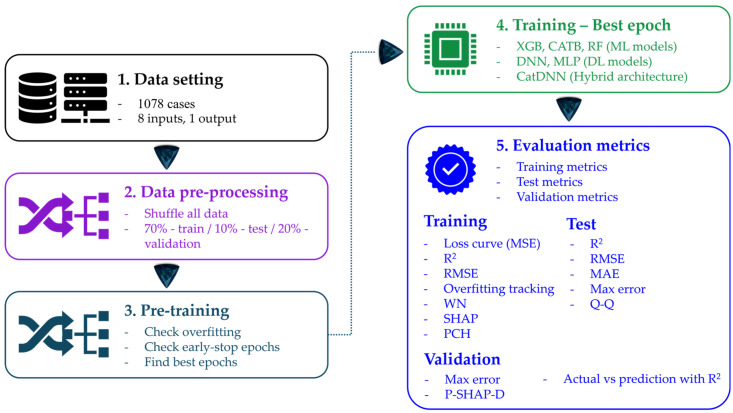
Total process of this study.

This study consists of 5 steps. Step 1 is dataset collection, Step 2 is data pre-processing, Step 3 is pre-training, Step 4 is training at the best epoch, and Step 5 is evaluation metrics. All the results presented in this study were obtained through these 5 steps, and the overall process is depicted in Figure 1.

In training, the loss function was the mean square error (MSE), which is the most representative training loss in DL studies. During training, R^2^, root mean square error (RMSE), overfitting tracking (OT), and weight norm (WN) were monitored. After training and before testing, the parameter relationships were evaluated by Shapley additive explanations (SHAP) and parameter correlation heatmap (PCH). Before training, datasets were divided into training for 70%, testing for 10%, and validation for 20%.

In testing, test R^2^, RMSE, mean absolute error (MAE), and max errors were evaluated to assess the training quality state. In addition to assessing the training quality, the quantile–quantile(Q-Q) relationship was analyzed to secure the test performance.

In validation, prediction for the validation parts was carried out. Partial SHAP dependence (P-SHAP-D) analysis was additionally performed. This study was concluded by validating the models through a comparative evaluation between the actual data and the predicted data (AvP), which is the most essential component of the validation process.

The optimizers for the NN-based models were implemented using the Adam algorithm provided by PyTorch version 2.0.1. The hyperparameters for the NN-based model were configured with a learning rate of 0.005, a full batch size, a mean squared error loss function, and an exponential linear unit activation function.

### 2.2. Models

#### 2.2.1. XGB

XGB performs sequential learning based on an additive model structure, where each stage compensates for the errors of the previous one. In particular, it uses second-order derivative information optimization, and its built-in regularization process increases training speed while controlling overfitting. These characteristics were considered suitable for CPD estimation with multiple parameters, and therefore, XGB was employed in this study.

For training of XGB, hyperparameters were set as 300 boosting iterations, 6 tree depth, and 0.05 learning rate.

#### 2.2.2. CATB

CATB introduces the ordered boosting method to address the prediction shift problem commonly observed in traditional gradient boosting models. It minimizes potential data leakage in the processing of categorical variables through ordered target statistics. In addition, the use of symmetric trees helps control model complexity, prevent overfitting, and optimize prediction speed. These characteristics were considered suitable for CPD prediction results.

For training of CATB, hyperparameters were set as 300 boosting iterations, 6 tree depth, and 0.05 learning rate.

#### 2.2.3. RF

RF is an ensemble model that trains multiple DTs in parallel through the bagging method, and each node split is performed using an independent random selection of variables. This mechanism reduces the correlation between trees and improves the generalization ability of the model, resulting in robust prediction performance even in data environments that contain noise. In addition, the model provides feature importance values during the training process. These properties are the reason that RF can be used as a major ML model.

For training of RF, hyperparameters were set as 300 decision trees, 10 tree depth, and auto mode for the feature selection method.

#### 2.2.4. MLP

**Figure 2:**
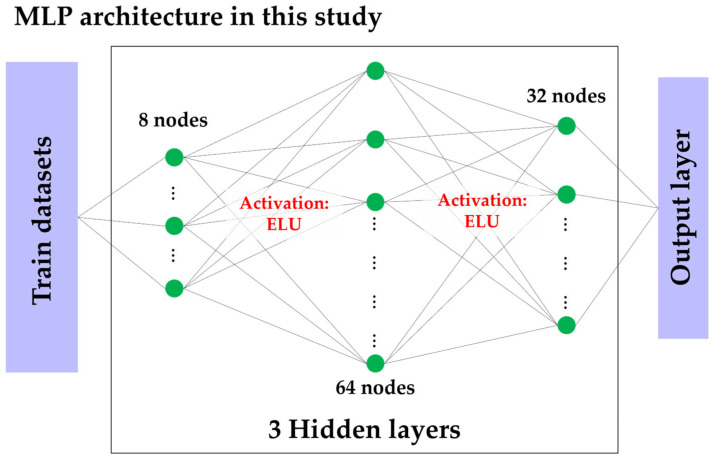
MLP architecture in this study.

MLP architecture was adopted in this study to learn the complex nonlinear relationships between concrete parameters and environmental factors. The input layer consists of eight independent variables, including W/C, cement content, and exposure duration, which have a dominant influence on chloride penetration. The hidden layers use the exponential linear unit (ELU) activation function to prevent the dying phenomenon of the rectified linear unit and to maintain activation values close to zero, thereby ensuring stable training. The output layer has a single node that produces the predicted CPD, and the optimal weights are obtained using the MSE loss function and the ADAM optimizer from PyTorch. Early stopping (ES) was also applied by monitoring the loss of the validation data to maximize generalization performance and minimize overfitting. The structure of the MLP used in this study is shown in Figure 2.

#### 2.2.5. DNN

**Figure 3:**
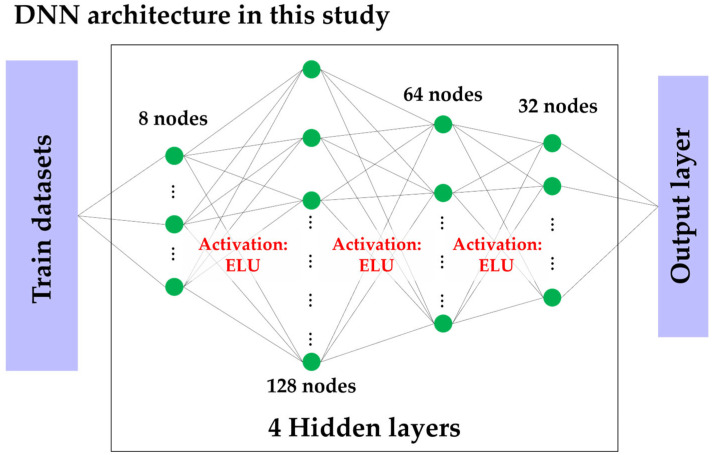
DNN architecture in this study.

DNN is an extended model of the MLP, with a deeper hidden-layer structure. The DNN architecture used in this study includes multiple hidden layers with 128, 64, and 32 nodes, respectively, aiming to extract complex and detailed features from the input data in a hierarchical manner. The training process applied the same ELU activation function and ADAM optimization algorithm used in the MLP, and ES was employed to prevent overfitting and ensure optimal generalization performance. The structure of the DNN used in this study is shown in Figure 3.

#### 2.2.6. CatDNN

**Figure 4:**
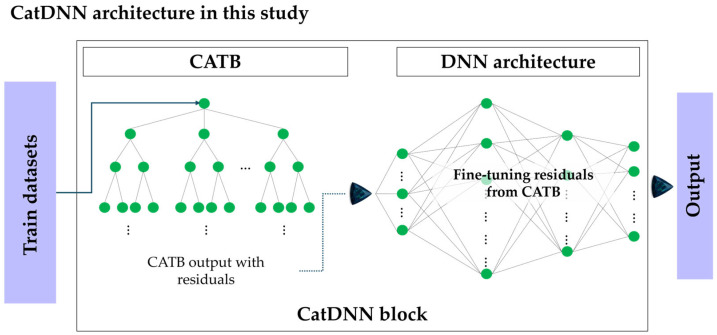
CatDNN architecture in this study.

In this study, a CatDNN hybrid architecture is proposed by combining the numerical advantages of the tree-based ensemble model CATB and the DNN. The model is based on a stage-wise learning mechanism. First, the CATB model learns the overall trend of the data and provides a baseline prediction. Then, the DNN is designed to learn the residuals, that is, the errors between the CATB output and the experimental values, in order to refine the fine-scale nonlinear relationships. The final prediction is obtained by mathematically summing the stable baseline output from CATB and the residual correction produced by the DNN. Theoretically, this hybrid approach can lead to a dramatic improvement in performance [22]. However, this study places greater emphasis on examining data saturation under a limited dataset and whether such limitations can be overcome. The structural flow of the CatDNN used in this study is shown in Figure 4.

### 2.3. Data Collection

The dataset used in this study was compiled from a total of 107 published references, and the corresponding sources are summarized in Table A1 of Appendix A. In addition, to ensure data reliability, the maximum–minimum ranges of each parameter are summarized in Table A2 of Appendix A.

### 2.4. Model Evaluation Metrics

#### 2.4.1. MSE Loss

In this study, MSE was used as the main loss function to quantitatively evaluate the training efficiency and prediction accuracy of the proposed models. MSE is calculated by squaring and averaging the difference between the experimentally obtained chloride penetration depth ($y_{i}$) and the predicted value (${\hat{y}}_{i}$) and is defined as Equation (1).

(1) $$MSE=\frac{\sum_{i=1}^{n}{\left(y_{i}-{\hat{y}}_{i}\right)}^{2}}{n}$$

where n is the number of samples. Because MSE calculates the squared error, it assigns a larger penalty to outliers where the difference between the actual and predicted values is large. This helps minimize critical prediction errors that may occur in concrete durability design and guides the model to converge stably toward a global minimum.

#### 2.4.2. R^2^

R^2^ is a statistical measure that indicates how accurately a regression model explains the overall variability of the dependent variable, the CPD. It represents the proportion of the total variance in the actual data that is explained by the model and is used as an indicator of the performance of the model of fit. The calculation of the coefficient of determination is performed using Equation (2).

(2) $$R^{2}=1-\frac{\sum_{i=1}^{n}{\left(y_{i}-{\hat{y}}_{i}\right)}^{2}}{\sum_{i=1}^{n}{\left(y_{i}-\bar{y}\right)}^{2}}$$

where $\bar{y}$ is the average of the samples. The numerator, $\sum_{i=1}^{n}{\left(y_{i}-{\hat{y}}_{i}\right)}^{2}$, represents the residual sum of squares that the model does not explain, while the denominator, $\sum_{i=1}^{n}{\left(y_{i}-\bar{y}\right)}^{2}$, represents the total sum of squares, which is the overall variability of the actual data. R^2^ takes a value between 0 and 1, and a value closer to 1 indicates a higher consistency between the regression line of the model and the observed data. In this study, R^2^ was used as a primary performance metric to compare the predictive performance of the proposed single machine learning algorithms and the hybrid CatDNN model under the same statistical criteria, and to verify how reliably each model architecture captures the nonlinear relationships between mixture parameters and environmental variables.

#### 2.4.3. ES

ES is a regularization technique introduced to prevent overfitting, which occurs when a model becomes excessively fitted to the training data during iterative learning. As the number of epochs increases, the error on the training data continues to decrease, but once the critical point is exceeded, the generalization performance of the model deteriorates, and the predictive accuracy for unseen data declines.

In this study, the loss values of a separate validation set were monitored in real time at every epoch to determine the stopping point of training. Specifically, when the validation loss no longer decreased and instead stagnated or increased for a predefined number of iterations (patience), the training process was forcibly terminated, and the weights corresponding to the minimum validation loss were saved as the final model. Through this mechanism, the MLP, DNN, and CatDNN models proposed in this study maintained a stable learning state that prevented unnecessary computational cost and ensured optimal generalization performance even under data saturation conditions. The patience value was set to 30, and the ES point was regarded as the best epoch. In the pre-training stage, 300 epochs were observed, and the subsequent full training of all the models was performed using the best epoch. All the criteria were based on the values obtained from CatDNN.

#### 2.4.4. Overfitting Monitoring

In this study, OT and WN were jointly used to evaluate the stability of model training and to precisely control overfitting. OT was used as an indicator to monitor the loss gap between the training dataset and the validation dataset in real time, allowing assessment of whether the model maintained sufficient generalization ability without becoming biased toward the training data. In parallel, WN was observed to quantify the structural complexity of the model. WN is defined as the square root of the sum of the squared weights (w) in the model (${||w||}_{2}$ = $\sqrt{\sum w_{i}^{2}}$, ${||w||}_{2}$ = WN), representing the overall magnitude of the neural network parameters. In general, an excessively large WN indicates that the model has learned local noise in the training data, resulting in unnecessarily high complexity.

Therefore, in this study, the expansion of the loss gap was monitored through OT, while the abnormal growth of the weights was suppressed through WN. This multi-faceted monitoring framework guided the neural networks to learn the general physical relationships between parameters and CDP in a stable manner and ultimately provided the basis for constructing a reliable hybrid model even under data saturation conditions.

#### 2.4.5. Parameter Correlation

The SHAP technique was introduced to address the inherent opacity of the artificial intelligence models and to provide engineering reliability for the prediction results. SHAP is based on the Shapley value from cooperative game theory and decomposes the influence of each input parameter on a specific prediction in an additive manner. By identifying the positive or negative contribution of each input variable to the final output, SHAP was used as a statistical basis for interpreting how the data-driven model reflects the correlations among input parameters.

In addition, PCH was used as an analytical tool to examine the complex dependencies among input variables. In this study, PCH represents the visualization of the correlation coefficients among the SHAP values of each input variable. This goes beyond the linear relationships present in the raw data and indicates how input parameters interact statistically within the internal computational process of the model to contribute to the output. Consequently, the combined use of SHAP and PCH served as an objective interpretive framework demonstrating that the proposed hybrid model systematically captures multidimensional interactions among variables.

#### 2.4.6. Test Performance Evaluation

After the training of each model proposed in this study was completed, an independent test dataset that was not included in the training process was used to comprehensively evaluate the generalization performance and prediction accuracy of the models. To quantify model performance from multiple perspectives, MAE, RMSE, and Max error were introduced as evaluation metrics in addition to R^2^.

First, MAE represents the arithmetic mean of the absolute differences between ${\hat{y}}_{i}$ and $y_{i}$, providing an intuitive measure of the average magnitude of error. MAE is calculated using Equation (3).

(3) $$MAE=\frac{\sum_{i=1}^{n}\left|y_{i}-{\hat{y}}_{i}\right|}{n}$$

In parallel, RMSE was computed to capture the sensitivity to error variability. RMSE is obtained by taking the square root of the mean of the squared errors, assigning greater weight to larger deviations and serving as an indicator of prediction stability. RMSE is calculated using Equation (4).

(4) $$RMSE=\sqrt{\frac{\sum_{i=1}^{n}{\left(y_{i}-{\hat{y}}_{i}\right)}^{2}}{n}}$$

Additionally, Max error was examined to identify the worst prediction failure that the model may produce for a specific combination of input parameters. Max error represents the maximum error observed within the dataset and provides essential information for defining the reliability limits of the model. Max error is calculated using Equation (5).

(5) $$Max\;error=\max\left(\left|y_{i}-{\hat{y}}_{i}\right|\right)$$

Beyond numerical indicators, Q-Q analysis was performed to verify whether the models systematically learned the overall distribution of the data. Q-Q analysis compares the distribution of the actual observations with that of the predicted values and visualizes how closely each quantile aligns with the 45-degree ideal line. If the data points are linearly aligned along this reference line, it statistically demonstrates that the model successfully captures the probabilistic relationship between the input parameters and the target variable without bias toward specific regions.

By integrating the numerical metrics, including R^2^, with the distributional validation provided by Q-Q analysis, the reliability of the proposed CatDNN and the comparison models was rigorously evaluated.

#### 2.4.7. Validation Performance Evaluation

P-SHAP-D and AvP analyses were sequentially performed to examine the internal learning validity of the constructed models and to ensure the reliability of the final predictions. First, P-SHAP-D analysis was conducted to identify the output tendencies of the model in response to numerical variations in the input parameters. In particular, the analysis focused on the top 3 most sensitive parameters, which were selected based on their dominant influence on the model predictions. P-SHAP-D visualizes how the model interprets the influence of these key variables across the entire data domain by mapping the variation in each selected parameter to the corresponding changes in SHAP values. Through this process, the analysis statistically verified whether the model successfully learned consistent data patterns associated with increases or decreases in the major input parameters, beyond simple numerical matching.

After confirming the internal learning behavior of the model, AvP analysis was performed as the final step to evaluate the agreement between the actual observations and the model predictions for the test dataset. The numerical precision of the model was assessed by examining whether all the data points were concentrated within a narrow error range around the identity line of the form y = x. By integrating the validation of key variable logic through P-SHAP-D with the precision assessment provided by AvP, the proposed model was demonstrated to possess sufficient statistical reliability and general applicability as a data-driven prediction model.

## 3. Results and Discussion

### 3.1. Pre-Training Results

#### Loss and ES Points

**Figure 5:**
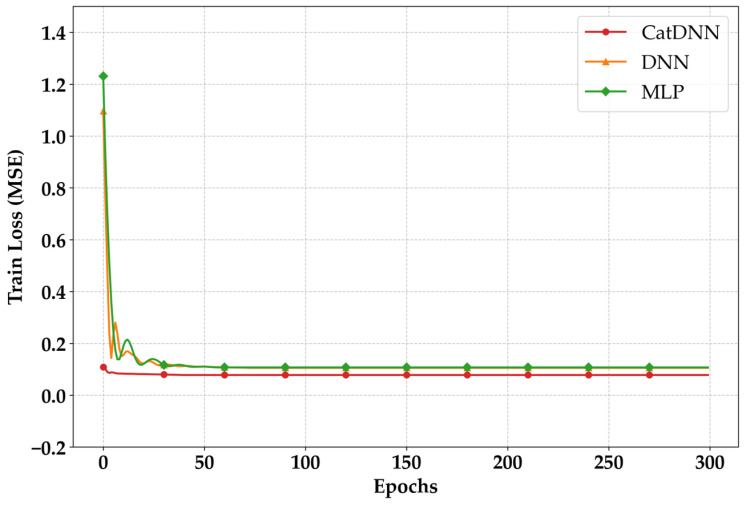
Pre-training loss.

Figure 5 indicates the pre-training loss results of NN models. The loss tendencies of the NN models become excessively flat after 50 epochs, indicating that the models are becoming overfitted. If the uncertainty range becomes excessively narrowed due to overtraining, the model loses its flexibility to predict the data accurately. In other words, the prediction performance deteriorates. In short, this behavior explains the necessity of the ES point.

**Figure 6:**
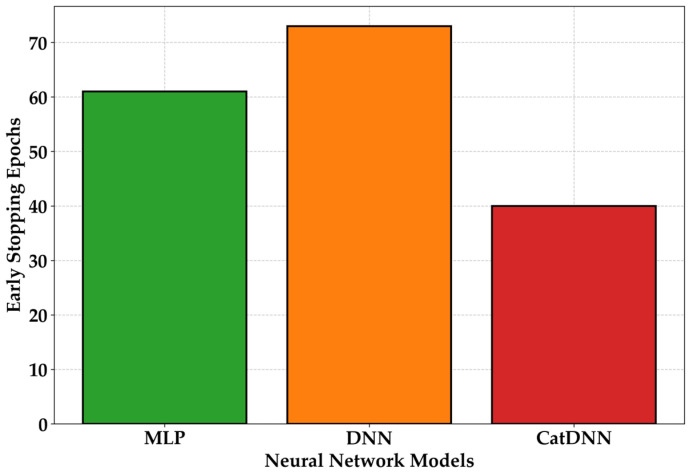
ES results.

The ES results monitored during the pre-training process are shown in Figure 6. The ES points were 61 for MLP, 72 for DNN, and 40 for CatDNN, indicating that CatDNN stabilized the earliest. Therefore, the best epoch in this study was determined to be 40. The main training was conducted using 40 epochs.

### 3.2. Main Training Results

**Figure 7:**
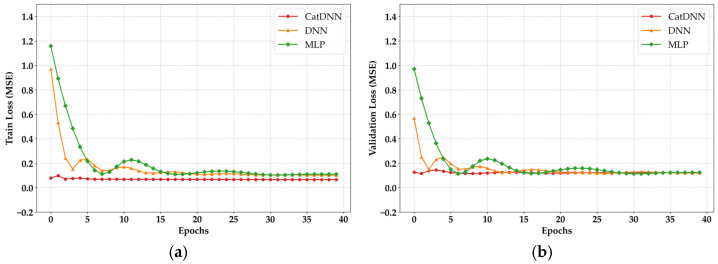
Loss results. (**a**) Training loss; (**b**) Validation loss.

Figure 7 indicates the training loss results of NN models. Although XGB and CATB include iterative boosting procedures, their optimization mechanisms differ from the gradient-based backpropagation used in neural network models [24,25]. Specifically, tree-based models focus on recursive partitioning of the feature space rather than continuous weight updates. Therefore, the notion of loss in these models reflects the reduction in impurity or residuals at each stage, rather than the convergence of a global error function as observed in neural network training. In addition, RF is an ensemble model composed of independent decision trees, and unlike boosting or neural network models, it does not involve an iterative loss-convergence process [26].

Because MLP has a shallow structure, its training stabilized later than DNN and CatDNN, occurring after approximately 30 epochs. However, in the validation loss, both MLP and DNN showed unstable behavior until before 30 epochs. The dataset of 1078 cases is sufficiently large for DL research. The unstable patterns observed in the early training and validation stages are considered to result from the compact architectures of MLP and DNN. If the hidden layers had been configured uniformly, as in early DL studies, the models would likely have shown slightly more stable behavior in the initial training phase [27]. However, considering the sensitivity to overfitting in recent research, the architecture adopted in this study is appropriate and does not present any issues.

**Figure 8:**
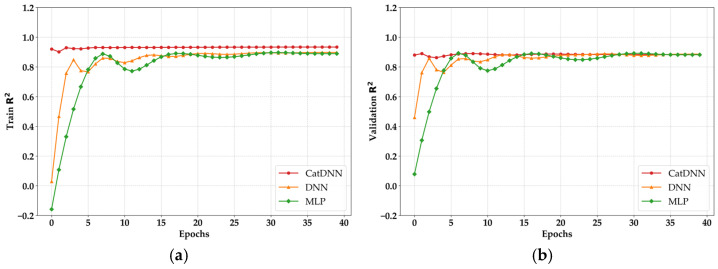
R^2^ monitoring results. (**a**) Training R^2^; (**b**) Validation R^2^.

The most distinctive result in Figure 7 is observed in CatDNN. From the early stage of training, both loss curves show almost no fluctuation. This behavior originates from CATB. Many studies using datasets similar to those in this research have reported comparable tendencies [28]. CATB is a model that performs strongly with categorical structures, meaning it is specialized in classifying data and determining the best decision [24]. In CatDNN, CATB produces near-optimal predictions for most cases before the residuals are passed to the DNN. As a result, the loss computed in the DNN component is smaller than that of the other NN models. Furthermore, adjusting the weights to correct residual errors is not a difficult task for DNN. The learning state cannot be evaluated solely based on the loss results. As shown in Figure 8, both training R^2^ and validation R^2^ were monitored simultaneously during the training process.

The R^2^ results showed tendencies similar to the loss curves. Before 30 epochs, both DNN and MLP exhibited unstable behavior. In addition, CatDNN maintained stable R^2^ values after early stabilization, consistent with its loss pattern. This tendency was observed in both the training and validation processes. Most importantly, all the models achieved R^2^ values exceeding 0.8, and in general, an R^2^ value above 0.8 is considered to indicate very high reliability for the corresponding model [29,30].

Although the training of the models can be considered sufficiently completed, overfitting cannot be assessed solely from the tendencies of the loss and R^2^ curves. At the ES point introduced to prevent overfitting, a duration of 40 epochs is long enough for overfitting to occur. Furthermore, although 1078 cases may be adequate for training, this quantity is very small compared to image-based learning tasks that commonly use datasets of twenty thousand or even one hundred thousand samples [31,32]. Therefore, data saturation may occur.

**Figure 9:**
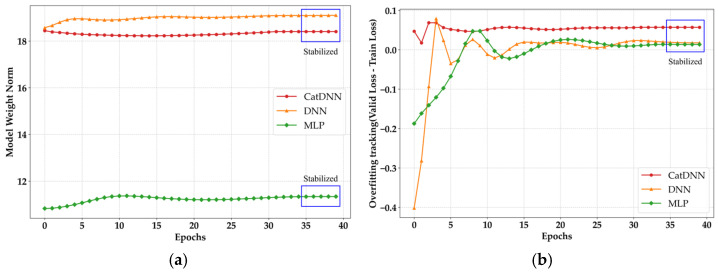
Overfitting monitoring results. (**a**) WN results; (**b**) OT results.

Figure 9 presents the monitoring results of WN and OT. In all the neural network models, WN converged to a specific value as training progressed and formed a flat and stable region. In general, when a model becomes excessively fitted to local noise in the training data, the magnitude of the weight increases abnormally, causing a sharp rise in the WN value [23]. However, all the models in this study maintained stable WN values, indicating that the structural complexity of the models was properly controlled without abnormal weight inflation. In the OT results, a somewhat concerning pattern can be observed. Due to the limited amount of data, CatDNN shows a flat tendency around 40 epochs. An excessively stabilized OT curve may not necessarily indicate overfitting, but it is sufficient to raise suspicion of data saturation. This suggests that the model is no longer improving and has become fixed due to data saturation.

**Figure 10:**
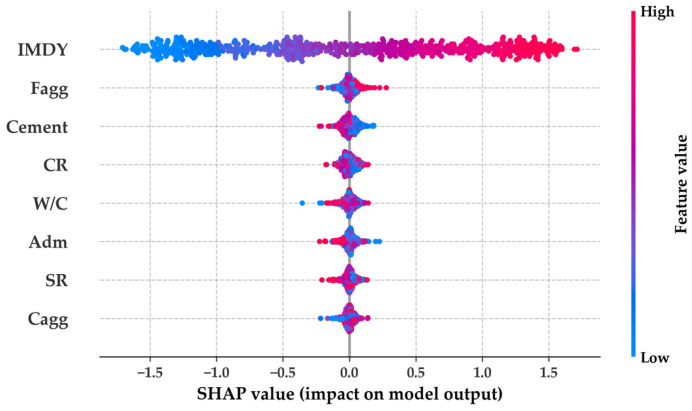
SHAP results.

**Figure 11:**
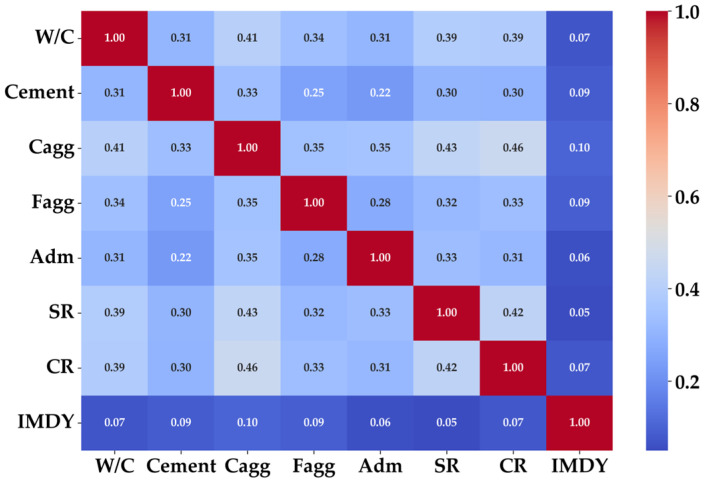
PCH analysis results.

To investigate the absence of performance improvement despite increased structural complexity, analyses corresponding to Figure 10 and Figure 11 were conducted. The SHAP summary plot in Figure 10 indicates that IMDY is the most influential variable across all the models, followed consistently by Cement and Fagg. The hybrid CatDNN architecture does not exhibit notable differences in variable-level contribution patterns compared with the standalone DNN or CATB models. This observation suggests that architectural refinement does not expand the range of extractable features beyond the informational limits of the dataset.

The PCH analysis in Figure 11 further shows that certain pairs of input variables display very high correlations in their SHAP values. This pattern implies that model predictions are dominated by interdependent or redundant information rather than independent variable contributions. The inability of the CatDNN residual correction mechanism to extract information beyond that captured by the individual models provides strong evidence that the statistical flexibility of the training dataset has reached saturation.

Overall, the consistent information structure and strong variable interdependencies indicate that increased data diversity is more critical than additional architectural complexity. These findings offer qualitative support for the data saturation hypothesis proposed in this study.

### 3.3. Test Results

The performance of each model was examined immediately after training, and the test results are summarized in Table 1. The ML and DL models used in this study are all models known to exhibit powerful performance in general research. As a result, RMSE, MAE, and max error related to the test errors showed only minor differences. In other words, the differences reported in Table 1 can be regarded as comparable from a practical engineering perspective. However, one notable outcome is that CatDNN showed a max error of 1.21, which is relatively distinct from the other models. This indicates that the principle of residual learning, which aims to convert false predictions into values closer to true predictions, functioned as intended in this study. Nevertheless, the R^2^ values do not show significant improvement. The R^2^ of CatDNN does not differ meaningfully from those of DNN/MLP, and CatDNN even shows a slightly lower R^2^ than DNN. This is considered to reflect the symptoms of data saturation that were previously noted in Figure 9b. More detailed conclusions must be drawn through validation.

### 3.4. Validation Results

**Figure 12:**
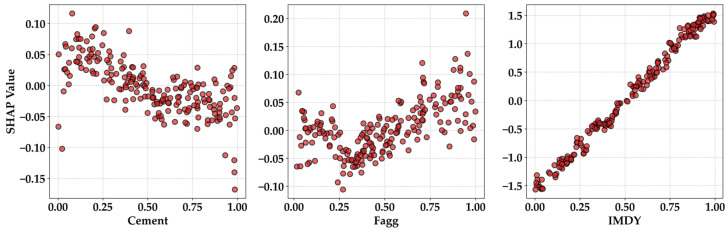
P-SHAP-D results.

According to Figure 10, the most sensitive factors affecting CPD are cement content, Fagg, and IMDY. IMDY is the most direct influencing factor for CPD [33]; therefore, the linear tendency observed in Figure 10 is reasonable. Cement content represents an increase in binder. In general, mortar tends to show lower CPD values than concrete for the same penetration age [34,35]. An increase in aggregate leads to an expansion of the interfacial transition zone and an increase in other voids. When viewed per unit volume, mortar contains a higher unit cement content than concrete. Therefore, as the mixture becomes closer to pure cement paste, chloride resistance improves [36]. The results related to cement in Figure 12 are reasonable. The results for Fagg show a weak V-shaped pattern. Fagg reflects the filler effect of fine particles. In other words, an appropriate amount of Fagg can fill the pores generated by the hydration of cement. Pores directly correspond to a reduction in strength, whereas filling by particles contributes to strength enhancement. Such findings have been reported in many studies [37,38,39].

In practice, a substantial portion of the volume in mortar or concrete is filled with aggregates for reasons of workability and economic efficiency, and as long as a certain strength level is secured, aggregates occupy the volume. This means that the application of aggregates according to standard construction guidelines significantly reduces the intrinsic performance of the cement paste. From this perspective, the Fagg results in P-SHAP-D represent a predictable behavior [40]. Thus, it was verified that the parameters, when used as inputs, were at least not applied in an incorrect direction.

**Figure 13:**
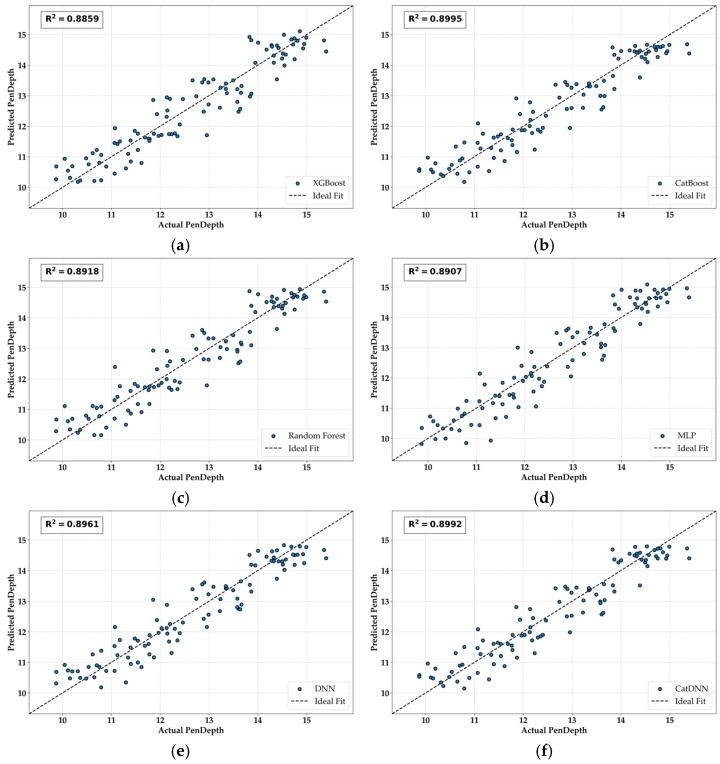
AvP results. (**a**) XGB; (**b**) CATB; (**c**) RF; (**d**) MLP; (**e**) DNN; (**f**) CatDNN.

Figure 13 shows the AvP results. All the models demonstrate sufficient competitiveness, with the validation R^2^ values exceeding 0.88. In addition, the R^2^ values do not show meaningful differences. CatDNN, which was expected to exhibit the best performance, showed results similar to those of DNN and CATB, and CATB even achieved a slightly higher R^2^ than CatDNN. In other words, due to data saturation, the less complex CATB model ultimately performed more efficiently. This result suggests that, for datasets with a structure and scale similar to those used in this study, applying a simple but well-fitted model may be more competitive than increasing model complexity.

The implementation of the developed models facilitates the advancement in digital durability assessment frameworks. These tools enable infrastructure managers to estimate CPDs rapidly, thereby optimizing maintenance schedules without exhaustive experimental testing. During the design phase, the predictive accuracy allows for the preliminary screening of concrete mixtures, significantly reducing material costs and laboratory validation time. Furthermore, the identified data saturation limits provide a pragmatic criterion for selecting model complexity based on available regional data scales. Integrating such data-driven architectures into structural health monitoring systems enhances the long-term sustainability and reliability of concrete structures in chloride-prone environments.

## 4. Conclusions

This study evaluated the prediction of CPD using three ML and three DL models—XGB, CATB, RF, MLP, DNN, and the hybrid CatDNN—based on a dataset consisting of eight input parameters (W/C, cement, Cagg, Fagg, Adm, SR, CR, and IMDY) and one output parameter (CPD). A total of 1078 data cases collected from 107 published references were used. Detailed conclusions are as follows:

(1) The CatDNN model exhibited the earliest stabilization during training, reaching its best epoch at approximately 40. However, OT and WN monitoring revealed a flat tendency near this point, indicating the onset of data saturation under limited data conditions. Simpler models such as CATB and DNN maintained stable learning behavior without abnormal weight growth.
(2) Test performance showed only marginal differences among all the models, with R^2^ = 0.8908~0.9129, RMSE = 0.4544~0.5088, and MAE = 0.3571~0.4037. Although CatDNN achieved the lowest maximum error (1.21) due to its residual learning mechanism, its R^2^ (0.9123) was slightly lower than that of DNN (0.9129), demonstrating that increased architectural complexity did not yield meaningful performance improvement.
(3) The validation results further confirmed this trend, with all the models achieving R^2^ higher than 0.88 and CATB outperforming CatDNN in some cases. P-SHAP-D analysis verified that the models learned physically consistent tendencies for the key parameters, particularly cement content, fine aggregate ratio, and immersion days.
(4) Overall, the findings demonstrate that when only limited datasets are available, simpler and well-fitted models can be more efficient and reliable than complex hybrid architectures. Future research should explore strategies to mitigate data saturation and enhance predictive accuracy under constrained data environments.

## Figures and Tables

**Table 1:** Test results.

Models	R^2^	RMSE	MAE	Max Error
XGB	0.8933	0.5029	0.4037	1.29
CATB	0.8973	0.4933	0.3964	1.28
RF	0.8908	0.5088	0.3879	1.37
MLP	0.9079	0.4672	0.3669	1.28
DNN	0.9129	0.4544	0.3571	1.35
CatDNN	0.9123	0.4561	0.3626	1.21

## Data Availability

The original contributions presented in this study are included in the article. Further inquiries can be directed to the corresponding author.

## Appendix A

**Table A1:** List of data sources for this study.

Authors	Literature Title	Link
Al Mamun et al.	Experimental Investigation of Chloride Ion Penetration and Reinforcement Corrosion in Reinforced Concrete Member	https://doi.org/10.6106/JCEPM.2017.3.30.026
Al Menhosh et al.	Sustainable high-performance concrete using metakaolin additive and polymer admixture: mechanical properties, durability and microstructure	https://tinyurl.com/dueya6du
Al-Ameeri et al.	Influence of carbonation on the resistance of concrete structures to chloride penetration and corrosion	https://doi.org/10.1051/matecconf/201928908001
Al-Ameeri et al.	Combined impact of carbonation and crack width on the Chloride Penetration and Corrosion Resistance of Concrete Structures	https://doi.org/10.1016/j.cemconcomp.2020.103819
Ali et al.	Improving the performance of recycled aggregate concrete using nylon waste fibers	https://doi.org/10.1016/j.cscm.2022.e01468
Ali et al.	Effect of sulfate activation of fly ash on mechanical and durability properties of recycled aggregate concrete	https://doi.org/10.1016/j.conbuildmat.2021.122329
Almeida et al.	Sugarcane bagasse ash sand (SBAS): Brazilian agroindustrial by-product for use in mortar	https://doi.org/10.1016/j.conbuildmat.2015.02.039
Almeida et al.	Use of sugarcane bagasse ash sand (SBAS) as corrosion retardant for reinforced Portland slag cement concrete	https://doi.org/10.1016/j.conbuildmat.2019.07.217
Althoey et al.	Sulfate activation of wheat straw ash to enhance the properties of high-performance concrete with recycled aggregates and waste tire steel fibers	https://doi.org/10.1371/journal.pone.0311838
Anssari et al.	The Influence of Calcined Alumina Additives on the Mechanical Properties and Chloride-Induced Corrosion of Blended Concrete	https://doi.org/10.56294/sctconf2024814
Bao et al.	Influence of the incorporation of recycled coarse aggregate on water absorption and chloride penetration into concrete	https://doi.org/10.1016/j.conbuildmat.2019.117845
Basu et al.	Permeation characteristics of self-compacting concrete containing sandstone slurry	https://doi.org/10.1016/j.matpr.2020.02.326
Beltrán et al.	Effect of cement addition on the properties of recycled concrete to reach control concretes strengths	https://doi.org/10.1016/j.jclepro.2014.05.053
Binici et al.	Effect of crushed ceramic and basaltic pumice as fine aggregates on concrete mortars properties	https://doi.org/10.1016/j.conbuildmat.2006.06.002
Binici et al.	Hydro-abrasive erosion of concrete incorporating ground blast-furnace slag and ground basaltic pumice	https://doi.org/10.1016/j.conbuildmat.2008.03.003
Caronge et al.	Feasibility study on the use of processed waste tea ash as cement replacement for sustainable concrete production	https://doi.org/10.1016/j.jobe.2022.104458
Chang et al.	The application of waste marble as coarse aggregate in concrete production	https://doi.org/10.24191/srj2018.15.1.75-83
Chao et al.	Effects of silica fume and steel fiber on chloride ion penetration and corrosion behavior of cement-based composites	https://doi.org/10.1007/s11595-013-0679-4
Charime et al.	Mechanical, durability and microstructure properties of eco-friendly sand concrete incorporating cane ash	https://doi.org/10.1016/j.jobe.2024.108801
Cheewaket et al.	Initial corrosion presented by chloride threshold penetration of concrete up to 10 year-results under marine site	https://doi.org/10.1016/j.conbuildmat.2012.07.061
Chen et al.	Effect of hydrophobic nanoparticle size on corrosion resistance of superhydrophobic mortar	https://doi.org/10.1016/j.conbuildmat.2025.140282
Chen et al.	Use of nano-silica sol in concrete: Performance and influence mechanisms	https://doi.org/10.1016/j.conbuildmat.2023.134582
Chiang et al.	Relation between the diffusion characteristic of concrete from salt ponding test and accelerated chloride migration test	https://doi.org/10.1016/j.matchemphys.2007.05.041
Chiker et al.	Sodium sulfate and alternative combined sulfate/chloride action on ordinary and self-consolidating PLC-based concretes	https://doi.org/10.1016/j.conbuildmat.2015.12.123
Chindaprasirt et al.	Influence of fly ash fineness on the chloride penetration of concrete	https://doi.org/10.1016/j.conbuildmat.2005.08.010
Chindaprasirt et al.	Mechanical properties, chloride resistance and microstructure of Portland fly ash cement concrete containing high volume bagasse ash	https://doi.org/10.1016/j.jobe.2020.101415
Chindaprasirt et al.	Effect of carbon dioxide on chloride penetration and chloride ion diffusion coefficient of blended Portland cement mortar	https://doi.org/10.1016/j.conbuildmat.2007.06.002
Chindaprasirt et al.	Resistance to chloride penetration of blended Portland cement mortar containing palm oil fuel ash, rice husk ash and fly ash	https://doi.org/10.1016/j.conbuildmat.2006.12.001
Ching et al.	Sustainable production of concrete with treated alum sludge	https://doi.org/10.1016/j.conbuildmat.2021.122703
Choudhary et al.	Permeation, corrosion, and drying shrinkage assessment of self-compacting high strength concrete comprising waste marble slurry and fly ash, with silica fume	https://doi.org/10.1016/j.istruc.2021.05.008
Ding et al.	Experimental study and simulation calculation of the chloride resistance of concrete under multiple factors	https://doi.org/10.3390/app11125322
Du et al.	A targeted approach of employing nano-materials in high-volume fly ash concrete	https://doi.org/10.1016/j.cemconcomp.2019.103390
Elfmarkova et al.	Determination of the chloride diffusion coefficient in blended cement mortars	https://doi.org/10.1016/j.cemconres.2015.06.014
El-Hassan et al.	Enhancing the chloride ion penetration resistance of concrete using metal–organic frameworks	https://doi.org/10.1016/j.cscm.2024.e03463
Farooq et al.	Influence of nylon fibers recycled from the scrap brushes on the properties of concrete: valorization of plastic waste in concrete	https://doi.org/10.1016/j.cscm.2022.e01089
Fjendbo et al.	Correlating the development of chloride profiles and microstructural changes in marine concrete up to ten years	https://doi.org/10.1016/j.cemconcomp.2022.104590
Fu et al.	Chloride profile characterization by electron probe microanalysis, powder extraction and AgNO_3_ colorimetric: A comparative study	https://doi.org/10.1016/j.conbuildmat.2022.127892
Gautam et al.	Robustness of self-compacting concrete incorporating bone china ceramic waste powder along with granite cutting waste for sustainable development	https://doi.org/10.1016/j.jclepro.2022.132969
Ghazy et al.	Resistance of concrete to different exposures with chloride-based salts	https://doi.org/10.1016/j.cemconres.2017.09.001
Güneyisi et al.	Comparative study on strength, sorptivity, and chloride ingress characteristics of air-cured and water-cured concretes modified with metakaolin	https://doi.org/10.1617/s11527-007-9258-5
Güneyisi et al.	Effect of initial curing on chloride ingress and corrosion resistance characteristics of concretes made with plain and blended cements	https://doi.org/10.1016/j.buildenv.2006.07.008
Han et al.	Electrochemical characterization of chloride ion transport in rubber concrete	https://doi.org/10.1016/j.jobe.2023.106658
Hashim et al.	Enhancing the sustainability, mechanical and durability properties of recycled aggregate concrete using calcium-rich waste glass powder as a supplementary cementitious material	https://doi.org/10.1016/j.scp.2025.101985
Higashiyama et al.	Efficiency of ground granulated blast-furnace slag replacement in ceramic waste aggregate mortar	https://doi.org/10.1016/j.cemconcomp.2013.12.014
Higashiyama et al.	Chloride ion penetration into mortar containing ceramic waste aggregate	https://doi.org/10.1016/j.conbuildmat.2012.01.018
Higashiyama et al.	Compressive strength and resistance to chloride penetration of mortars using ceramic waste as fine aggregate	https://doi.org/10.1016/j.conbuildmat.2011.05.008
Higashiyama et al.	A visual investigation on chloride ingress into ceramic waste aggregate mortars having different water to cement ratios	https://doi.org/10.1016/j.conbuildmat.2012.11.078
Hong et al.	Mix proportion design based on particle compact packing theory and research on the resistance of metakaolin to chloride salt erosion in UHPC cementitious system	https://doi.org/10.1016/j.conbuildmat.2024.137982
Horsakulthai et al.	Investigation on the corrosion resistance of bagasse-rice husk-wood ash blended cement concrete by impressed voltage	https://doi.org/10.1016/j.conbuildmat.2010.06.057
Hou et al.	Study of corrosion resistance of superhydrophobic hydrotalcite cement mortar in chlorine salt environment	https://doi.org/10.1016/j.conbuildmat.2024.137124
Hu et al.	Chloride migration in cement mortars with ultra-low water to binder ratio	https://doi.org/10.1016/j.cemconcomp.2021.103974
Huang et al.	Using RCPT determine the migration coefficient to assess the durability of concrete	https://doi.org/10.1016/j.conbuildmat.2018.02.109
Huang et al.	Using the chloride penetration depth obtained from RCPT to assess the permeability of concrete	https://doi.org/10.6119/JMST.202004_28(2).0005
Huang et al.	Determination of the chloride migration coefficient for interfacial transition zone in cement-based material with fly ash replacement	https://doi.org/10.1016/j.cemconcomp.2022.104558
Huynh et al.	Evaluation of the cementing efficiency factor of low-calcium fly ash for the chloride-penetration resistance of concretes: A simple approach	https://doi.org/10.1016/j.conbuildmat.2020.121858
Jaafar et al.	Chloride resistance behavior on nano-metaclayed ultra-high-performance concrete	https://doi.org/10.1051/matecconf/201710301023
Jain et al.	Effect of granite industry waste addition on durability properties of fly ash blended self-compacting concrete	https://doi.org/10.1016/j.conbuildmat.2022.127727
Jain et al.	Electrical impedance analysis based quantification of microstructural changes in concretes due to non-steady state chloride migration	https://doi.org/10.1016/j.matchemphys.2011.04.057
Jang et al.	Resistance of coal bottom ash mortar against the coupled deterioration of carbonation and chloride penetration	https://doi.org/10.1016/j.matdes.2015.12.074
Jeon et al.	Effects of nano-silica and reactive magnesia on the microstructure and durability performance of underwater concrete	https://doi.org/10.1016/j.powtec.2021.11.020
Kandasamy et al.	The effect of permeable formwork on durability and corrosion performance of concrete	https://doi.org/10.1016/j.cscm.2021.e00838
Kim et al.	Evaluation of durability of concrete substituted heavyweight waste glass as fine aggregate	https://doi.org/10.1016/j.conbuildmat.2018.06.221
Kumar et al.	Evaluation of mechanical and carbonation properties of self-compacting treated recycled coarse aggregates with two-stage mixing approaches	https://doi.org/10.1016/j.clwas.2022.100060
Liu et al.	Predicting the chloride diffusion in concrete incorporating fly ash by a multi-scale model	https://doi.org/10.1016/j.jclepro.2021.129767
Liu et al.	Understanding the effect of curing age on the chloride resistance of fly ash blended concrete by rapid chloride migration test	https://doi.org/10.1016/j.matchemphys.2017.05.011
Liu et al.	Experimental investigation on mechanical and piezoresistive properties of cementitious materials containing graphene and graphene oxide nanoplatelets	https://doi.org/10.1016/j.conbuildmat.2016.10.024
Ma et al.	Improved chloride transport resistance of cement mortar using quartz sands modified with MgAl-LDH	https://doi.org/10.1016/j.jobe.2023.107557
Maes et al.	Resistance of concrete and mortar against combined attack of chloride and sodium sulphate	https://doi.org/10.1016/j.cemconcomp.2014.06.013
Mala et al.	Effect of relative levels of mineral admixtures on corrosion resistance of cracked ternary cement blend concrete	https://doi.org/10.1080/21650373.2013.847810
Ming et al.	Improved chloride binding capacity and corrosion protection of cement-based materials by incorporating alumina nano particles	https://doi.org/10.1016/j.cemconcomp.2022.104898
Mittri et al.	Utilization of heat-treated ornamental stone processing waste as an addition to concretes to improve compressive strength and reduce chloride ion penetration	https://doi.org/10.1016/j.conbuildmat.2018.08.129
Moradllo et al.	Using micro X-ray fluorescence to image chloride profiles in concrete	https://doi.org/10.1016/j.cemconres.2016.11.014
Muduli et al.	Performance assessment of concrete incorporating recycled coarse aggregates and metakaolin: A systematic approach	https://doi.org/10.1016/j.conbuildmat.2019.117223
Nasiru et al.	Properties of cement mortar containing recycled glass and rice husk ash	https://doi.org/10.1016/j.conbuildmat.2021.123900
Oni et al.	The effect of cassava starch on the durability characteristics of concrete	https://doi.org/10.2174/1874149502014010289
Pablo-Calderón et al.	Exploring the detection of Cl^−^ penetration in portland cement mortars via surface electrical resistivity	https://doi.org/10.3390/ma16227123
Park et al.	Evaluation of concrete durability performance with sodium silicate impregnants	https://doi.org/10.1155/2014/945297
Peng et al.	Enhancing the corrosion resistance of recycled aggregate concrete by incorporating waste glass powder	https://doi.org/10.1016/j.cemconcomp.2022.104909
Qiao et al.	Chloride diffusion and wicking in concrete exposed to NaCl and MgCl_2_ solutions	https://doi.org/10.1061/(ASCE)MT.1943-5533.0002192
Rahbar et al.	High-strength concrete containing RHA and nano-CaCO_3_	https://doi.org/10.1049/mnl.2019.0281
Rasid et al.	Ground palm oil fuel ash and calcined eggshell powder as SiO_2_-CaO based accelerator in green concrete	https://doi.org/10.1016/j.jobe.2022.105617
Rattanachu et al.	Performance of recycled aggregate concrete with rice husk ash as cement binder	https://doi.org/10.1016/j.cemconcomp.2020.103533
Rerkpiboon et al.	Strength, chloride resistance, and expansion of concretes containing ground bagasse ash	https://doi.org/10.1016/j.conbuildmat.2015.10.140
Said et al.	Properties of concrete incorporating nano-silica	https://doi.org/10.1016/j.conbuildmat.2012.06.044
Saleh et al.	Durability assessment of industrial by-product and marine resource-based ultra-high-performance concrete	https://doi.org/10.1016/j.matpr.2023.07.270
Samimi et al.	Influence of pumice and zeolite on compressive strength, transport properties and resistance to chloride penetration of high strength self-compacting concretes	https://doi.org/10.1016/j.conbuildmat.2017.06.071
Shaikh et al.	Sustainability assessment of recycled aggregates concrete mixes containing industrial by-products	https://doi.org/10.1016/j.mtsust.2019.100013
Shin et al.	Mix design of concrete for prestressed concrete sleepers using blast furnace slag and steel fibers	https://doi.org/10.1016/j.cemconcomp.2016.08.007
Siddique et al.	Sustainable utilization of ceramic waste in concrete: Exposure to adverse conditions	https://doi.org/10.1016/j.jclepro.2018.10.231
Simčič et al.	Chloride ion penetration into fly ash modified concrete during wetting-drying cycles	https://doi.org/10.1016/j.conbuildmat.2015.04.033
Somna et al.	Effect of ground fly ash and ground bagasse ash on the durability of recycled aggregate concrete	https://doi.org/10.1016/j.cemconcomp.2012.03.003
Somna et al.	Effect of ground bagasse ash on mechanical and durability properties of recycled aggregate concrete	https://doi.org/10.1016/j.matdes.2011.11.065
Sørensen et al.	Chloride penetration into concrete-Comparison of results from field exposure tests and laboratory tests	https://doi.org/10.13140/RG.2.1.4088.2960
Söylev et al.	The investigation of the validity of a single k-value for fly ash in the range of W/C ratio critical for the standards	https://doi.org/10.1016/j.conbuildmat.2020.118284
Spiesz et al.	The apparent and effective chloride migration coefficients obtained in migration tests	https://doi.org/10.1016/j.cemconres.2013.02.005
Sujjavanich et al.	Synergistic effect of metakaolin and fly ash on properties of concrete	https://doi.org/10.1016/j.conbuildmat.2017.08.072
Thomas et al.	Recycling of waste tire rubber as aggregate in concrete: durability-related performance	https://doi.org/10.1016/j.jclepro.2015.08.046
Wang et al.	Study on the effect of plant extracts as low carbon green admixtures on the performance of cement mortar	https://doi.org/10.1016/j.cscm.2023.e02080
Wang et al.	Performances of concrete with binder and/or aggregates replacement by all-solid waste materials	https://doi.org/10.1016/j.jclepro.2024.141929
Wu et al.	The effect of nano-scale calcium stearate emulsion on the integral waterproof performance and chloride resistance of cement mortar	https://doi.org/10.1016/j.conbuildmat.2021.125903
Yang et al.	A rapid method to determine the chloride profile in cement-based materials by using electrical field	https://doi.org/10.1080/02533839.2011.591547
Yazıcı et al.	The effect of silica fume and high-volume Class C fly ash on mechanical properties, chloride penetration and freeze–thaw resistance of self-compacting concrete	https://doi.org/10.1016/j.conbuildmat.2007.01.002
Yoon et al.	Quantitative relationship between chloride penetration depth and hydraulic conductivity of concrete under hydrostatic pressure	https://doi.org/10.1061/(ASCE)MT.1943-5533.0004198
Younis et al.	Durability and mechanical characteristics of sustainable self-curing concrete utilizing crushed ceramic and brick wastes	https://doi.org/10.1016/j.cscm.2022.e01251
Zhang et al.	Experimental study on chloride penetration of the new-to-old concrete interface	https://doi.org/10.1016/j.conbuildmat.2024.135585
Zheng et al.	Concrete pore structure and performance changes due to the electrical chloride penetration and extraction	https://doi.org/10.1080/21650373.2015.1087891
Zheng et al.	Innovative and environmentally friendly MICP surface curing: Enhancing mechanical and durability properties of concrete	https://doi.org/10.1016/j.jclepro.2024.143962

**Table A2:** Maximum–minimum ranges of parameters.

Parameters	Maximum	Minimum
W/C	1.500	0.156
Cement	0.526	0.04499
Cagg	0.576	0
Fagg	0.700	0
Adm	0.0189	0
SR	0.312	0
CR	0.255	0
IMDY	3650	0.125
CPD	89	0.5799
